# Author Correction: Faster chiral versus collinear magnetic order recovery after optical excitation revealed by femtosecond XUV scattering

**DOI:** 10.1038/s41467-021-21915-9

**Published:** 2021-03-04

**Authors:** Nico Kerber, Dmitriy Ksenzov, Frank Freimuth, Flavio Capotondi, Emanuele Pedersoli, Ignacio Lopez-Quintas, Boris Seng, Joel Cramer, Kai Litzius, Daniel Lacour, Hartmut Zabel, Yuriy Mokrousov, Mathias Kläui, Christian Gutt

**Affiliations:** 1 Institut für Physik, Johannes Gutenberg-Universität Mainz, 55099 Mainz, Germany; 2 Graduate School of Excellence Materials Science in Mainz, 55128 Mainz, Germany; 3 Department Physik, Universität Siegen, Walter-Flex-Strasse 3, 57072 Siegen, Germany; 4 Peter Grünberg Institute and Institute for Advanced Simulation, Forschungszentrum Jülich and JARA, 52425 Jülich, Germany; 5 Elettra-Sincrotrone Trieste, 34149 Basovizza Trieste, Italy; 6 Institut Jean Lamour, UMR CNRS 7198, Université de Lorraine, 54506 Vandoeuvre-lès-Nancy, France; 7 Department of Physics, Ruhr-University Bochum, 44780 Bochum, Germany

**Keywords:** Magnetic properties and materials, Ferromagnetism, Magnetic properties and materials

Correction to: *Nature Communications* 10.1038/s41467-020-19613-z, published online 9 December 2020.

The original version of this Article omitted the following from the Acknowledgements:

We acknowledge the financial support from the Horizon 2020 Framework Programme of the European Commission under FET-Open Grant No. 863155 (s-Nebula).

This has now been corrected in both the PDF and HTML versions of the Article.

