# Supplementary material for: Faster chiral versus collinear magnetic order recovery after optical excitation revealed by femtosecond XUV scattering
Source: Nat Commun. 2020 Dec 9;11:6304. doi: 10.1038/s41467-020-19613-z (PMC7726566; doi:10.1038/s41467-020-19613-z)
Supplement: Supplementary file 1 — Supplementary Information [file 41467_2020_19613_MOESM1_ESM.pdf]

Supplementary

S1: Determination of material parameters and their temperature dependence by SQUID/VSM

Hysteresis loops were recorded by SQUID and VSM in order to determine the saturation magnetisation M_s as well as the effective anisotropy K_{eff} of the sample. The curves displayed in Fig. S1 show the out-of-plane (OOP) and in-plane (IP) hysteresis loops at 300 K. The effective perpendicular anisotropy K_{eff} was determined by the difference in the areas of the in-plane and out-of-plane hysteresis loops and corresponds to $K_u - \mu_0/2\ M_s^2$ with the uniaxial anisotropy K_u [1].

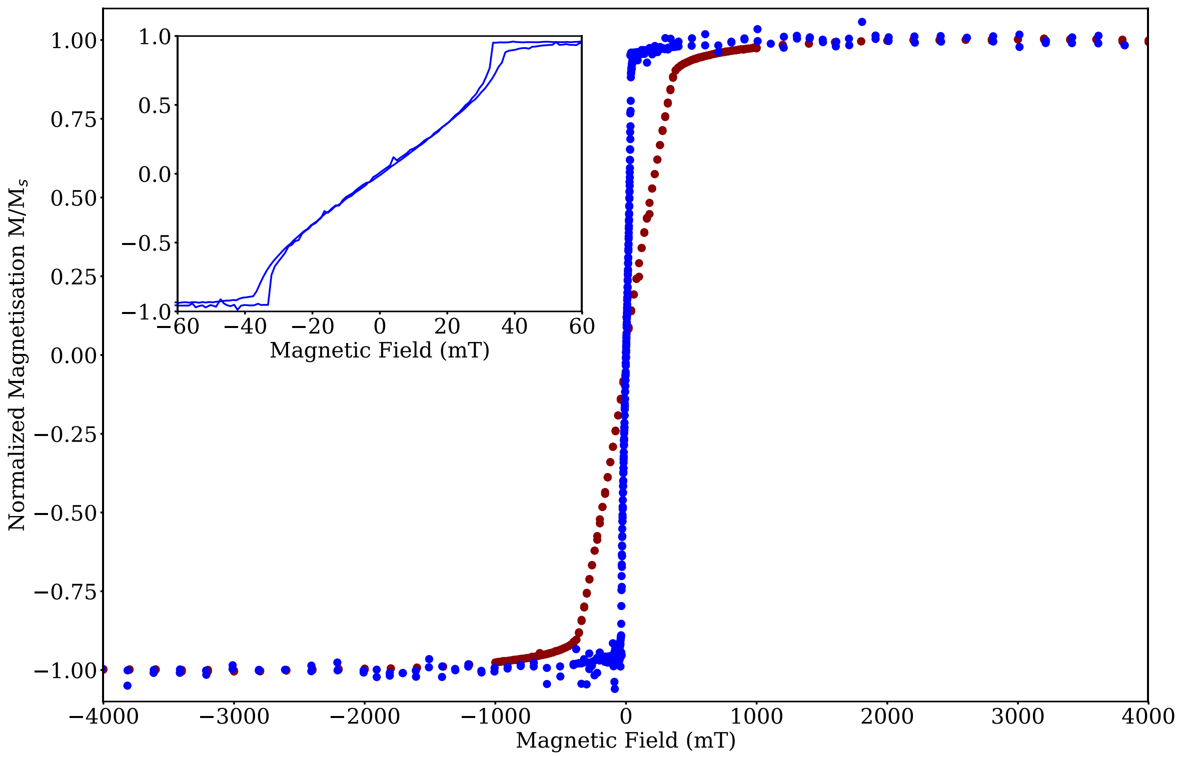

Figure S1: Hysteresis loops of multilayer stack. OOP (blue) and IP (red) hysteresis loops recorded at 300 K by SQUID magnetometry. Inset: The zoomed in OOP curve displays the typical behaviour of a multidomain stripe sample.

The hysteresis loops displayed in Fig. S1 measured at room temperature (300 K) via SQUID were also performed at temperatures of 10 K, 50 K, 100 K, 200 K, 300 K, 350 K and 390 K by SQUID and 300 K, 350 K, 390 K, 450 K, 500 K, 550 K, and 600 K by VSM. The saturation magnetisation was determined and afterwards the following model was used to fit the temperature dependence of the saturation magnetisation:

$$M_s(T) = M_s(0\ K)\ (1 - (T/T_c)^{\alpha})^{\beta}, \qquad (13)$$

with a saturation magnetisation M_s(0 K) = (976±10) kA/m, a Curie temperature T_c = (553±1) K, α = (1.73±0.13) and β = (0.63±0.04).

The temperature dependence of the effective anisotropy constant was fitted using:

$$K_{eff}(T) = K_u(T) - \frac{\mu_0}{2}(M_s(T))^2, \qquad (14)$$

where the magnetisation dependence of the anisotropy constant was fitted by a power law:

$$\frac{K_u(T)}{K_u(0\ K)} = \left(\frac{M_s(T)}{M_s(0\ K)}\right)^c, \qquad (15)$$

leading to an anisotropy constant $K_u(0\ K) = (1031 \pm 11)\ kJ/m^3$ and $c = (2.16 \pm 0.02)$.

Using the fit parameters, we obtain finally a room temperature saturation magnetisation of $M_s(300\ K) = (844 \pm 28)\ kA/m$ and an effective anisotropy of $K_{eff}(300\ K) = (133 \pm 17)\ kJ/m^3$**.**

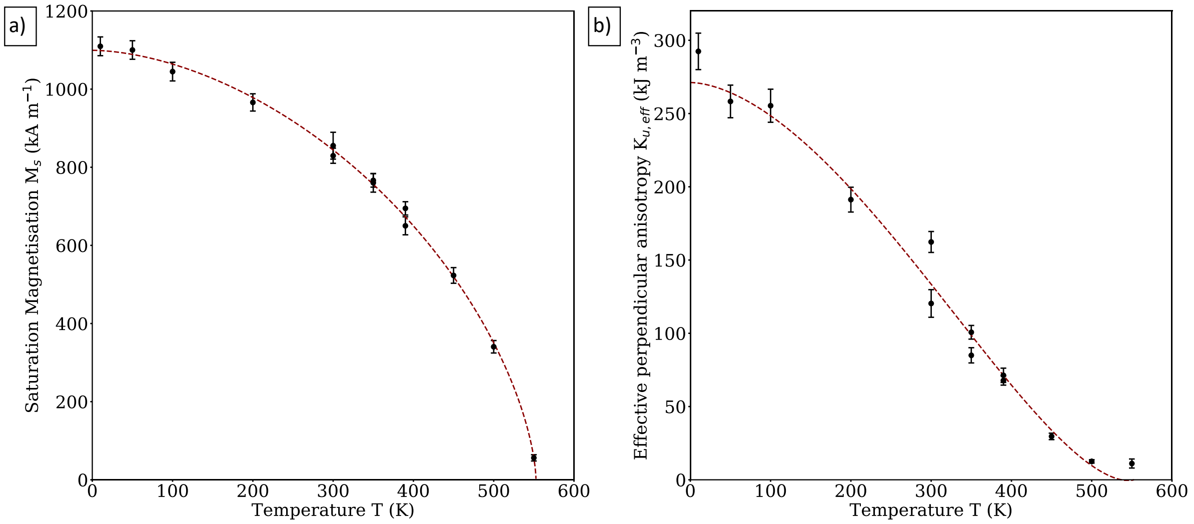

Figure S2: Temperature dependence of M_s and K_{eff}. Temperature dependence of the saturation magnetisation M_s (**a**) and effective anisotropy K_{eff} (**b**). The datapoints were fitted by the models displayed in Eq. (13) respectively Eq. (14).

S2: Pump-probe-delay time dependent spin system temperature

The total intensity of the sum signal displayed in Fig. 4a & 4b is connected to average domain magnetisation ($I \propto M^2$). Using the temperature dependence of the saturation magnetisation according to Fig. S2 we estimate the temperature evolution of the spin system during the pump-probe experiment displayed in Fig. S3. Upon optical excitation the spin system temperature increases within a ps to (493±22) K and relaxes afterwards slowly back to room temperature with an already decreased temperature of (469±13) K at 100 ps.

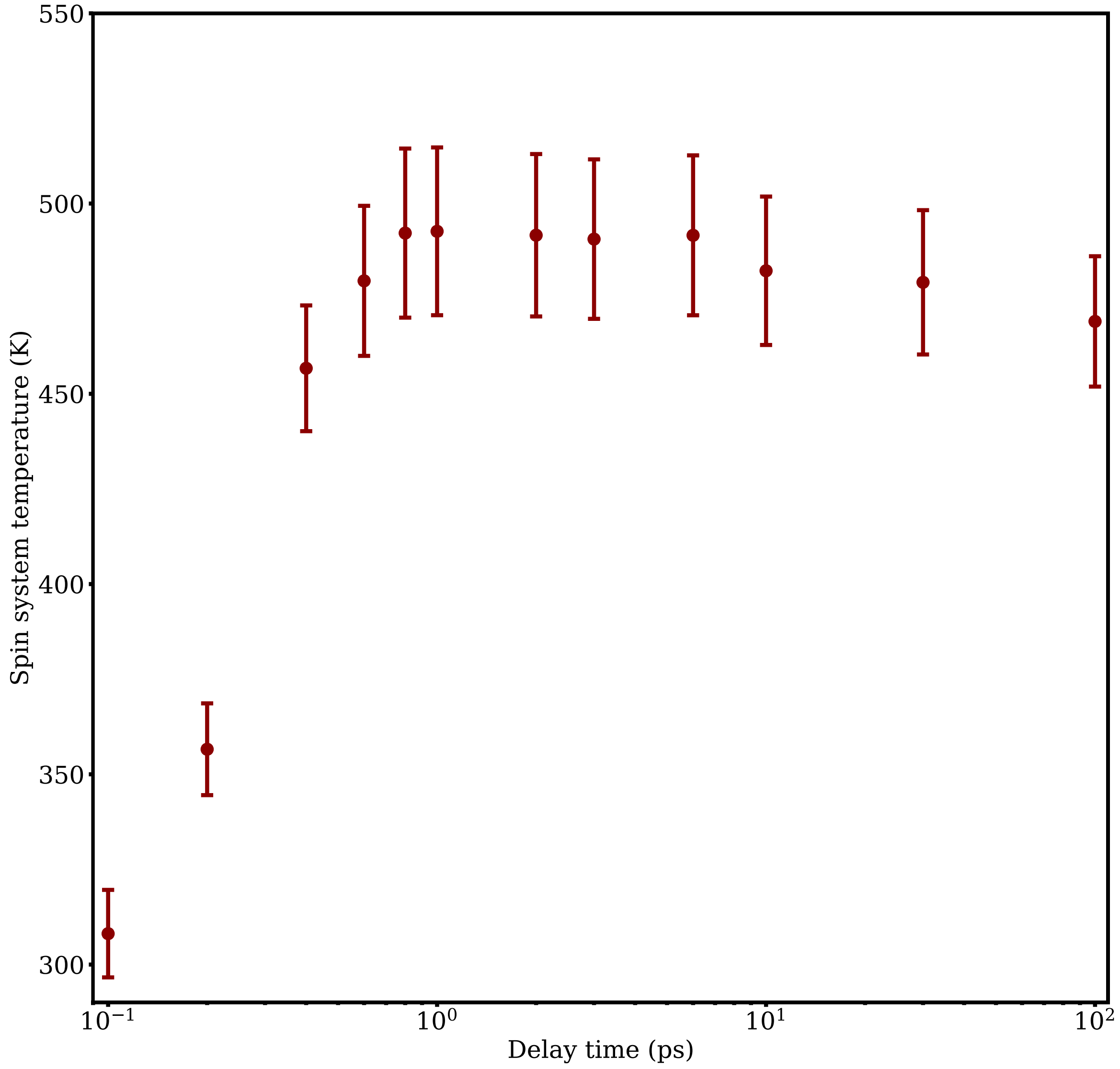

Figure S3: Time dependence of spin system temperature. The evolution of the spin system temperature as a function of pump-probe delay time in logarithmic scale.

S3: Micromagnetic simulations of the equilibrium domain wall configuration

Using MicroMagnum [2], a framework to perform numerical micromagnetic simulations, we simulated the complete [Ta(5.3 nm)/$Co_{20}Fe_{60}B_{20}$(0.93 nm)/Ta(0.08 nm)/MgO(2.0 nm)]$_{x20}$ multilayer stack using a cell size of 0.9 nm. The following material parameters have been used for the magnetic layers: the experimentally measured parameters M_s(300 K) = 844 kA/m and K_{eff}(300 K) = 133 kJ/m^3 , as well as the typical literature values of such material stacks for the DMI constant D = 0.06 mJ/m^2 [3-4] and the typically for CoFeB assumed exchange stiffness constant of A=10 pJ/m at room temperature [4]. The equilibration of a domain wall leads to the stabilization of the domain wall configuration as shown in Fig. S4. The exact domain wall arrangement is determined by an interplay between interfacial DMI and dipolar interactions that lead in the simulations to the stabilization of a so-called hybrid chiral domain wall [5]. The top and bottom magnetic layers in such a structure have opposite chirality due to flux closure, while the right-handed chirality is predominant in more layers due to the interfacial DMI provided by the Ta/CoFeB interface.

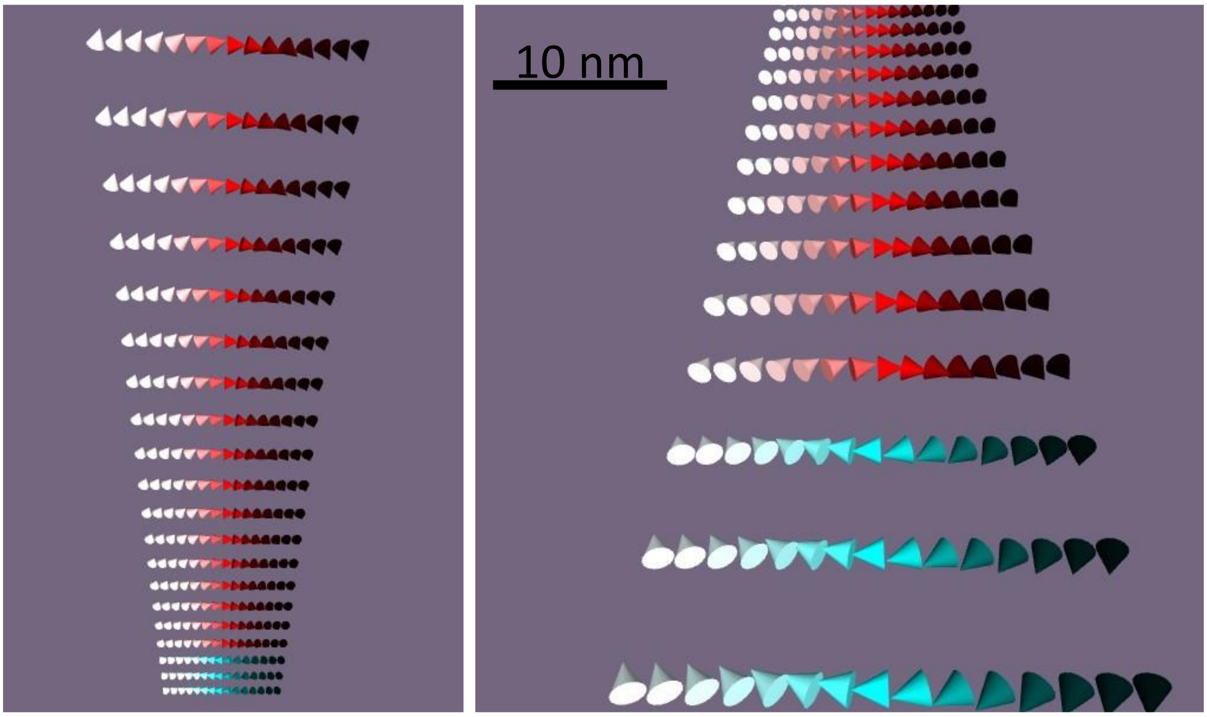

Figure S4: Domain wall configuration of the complete material stack. The magnetic moments of the individual cells of the different layers are depicted by the coloured arrows. While most layers are right-handed Néel type the three bottom layers are left-handed Néel type domain walls.

Analysing the domain wall profile in the simulations for the uppermost layer leads to a domain wall angle of φ = 90° degrees, supporting the experimental observation of fully right-handed Néel -type domain walls from the dichroic scattering signal.

By plotting the z-component of the magnetisation of the uppermost layer and using the expressions in Eq. (5) of the method sections, that are typically used to model homochiral domain walls, the domain wall width can be determined.

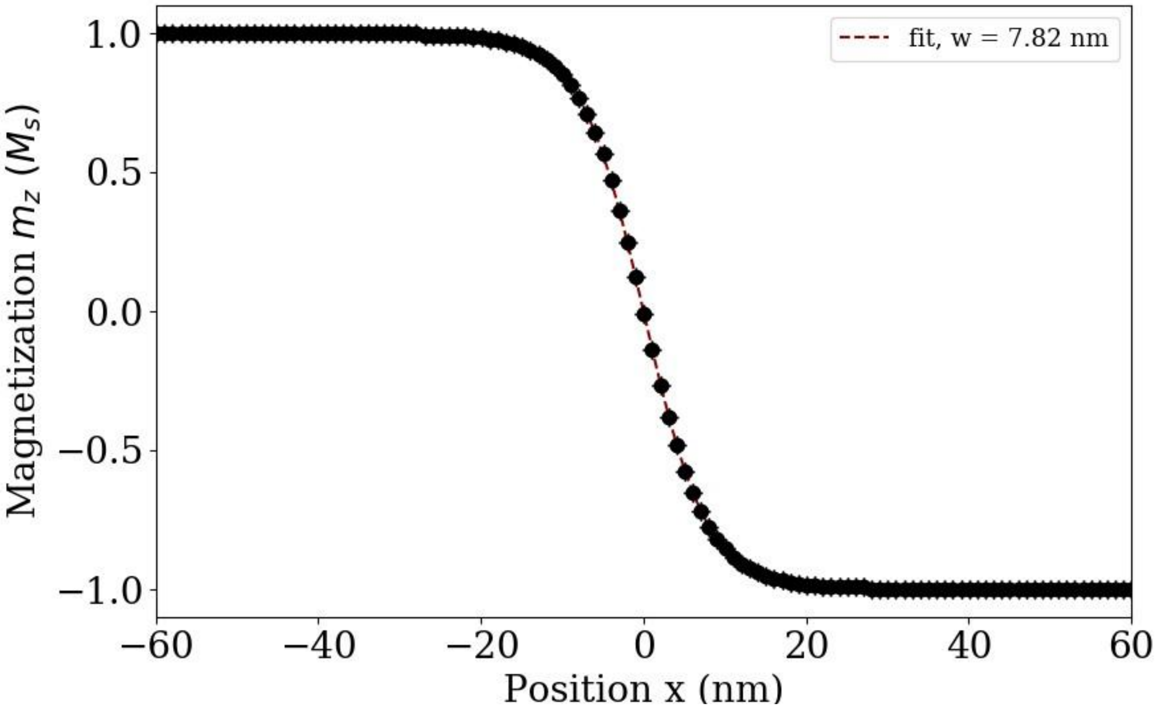

Figure S5: Magnetisation profile of a domain wall in the topmost magnetic layer from micromagnetic simulation. Fitting the profile of the z-component of the magnetisation leads to a domain wall width of approximately 8 nm.

Using the fit function $m_z^{W+-}(x) = -\tanh(\frac{x}{w})$, leads to a DW width of w = 7.82 nm ≈ 8 nm using the room temperature parameters.

This value is not differing significantly from the value obtained by the analytical formula:

$$\Delta = \sqrt{A/K_{u,eff}}\,, \quad (16)$$

typically used to determine the domain wall width from experimental material parameters [1]. Calculating the wall width with our material parameters leads to Δ = 8.6 nm. This shows that DMI as well as dipolar interactions contribute here strongly to the exact domain wall configuration [5], while the domain wall width is not affected significantly by the value of the DMI [4,6].

S4: Temperature dependence of the domain wall width

The domain wall width in these thin film samples is governed by Eq. (16) with no significant effect of the DMI [4,6]. Using the temperature dependence of the effective anisotropy constant obtained in Fig. S2 one can estimate the temperature dependence of the (equilibrated) domain wall width using Eq. (16).

The temperature dependence of the exchange stiffness constant A is in mean-field approximation described by a power law $A \propto M_s^2$ leading to the temperature scaling of the domain wall width displayed in Fig. S6. One can observe that the domain wall width increases with increasing temperature due to a stronger scaling of the effective anisotropy constant in comparison to the exchange constant. It was also shown that fluctuation corrections from nonlinear spin-wave effects lead to a scaling $A \propto M_s^\kappa$ with $\kappa<2$ depending on the lattice structure [7], which would lead to an even stronger scaling of the domain wall width with the temperature.

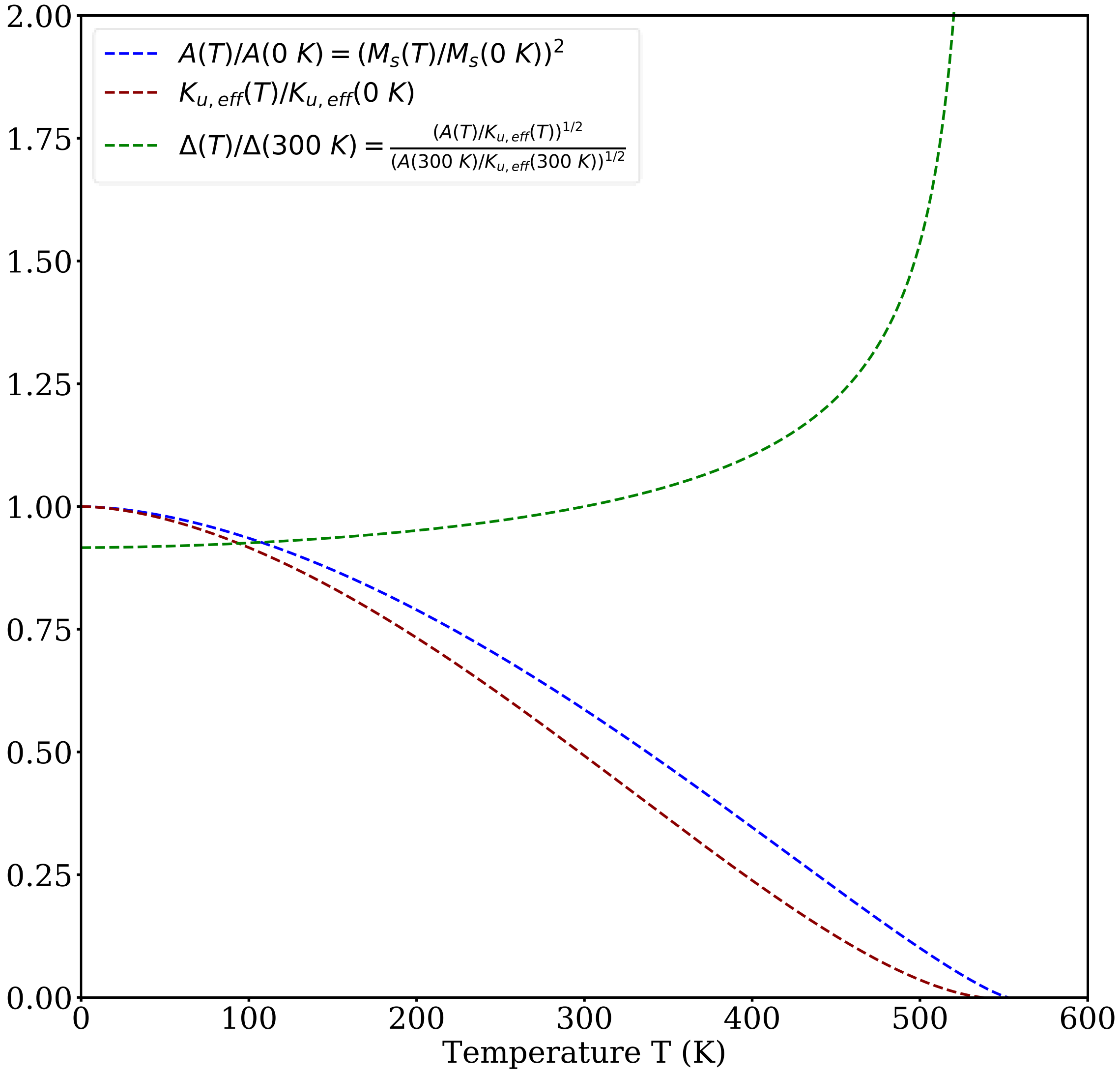

Figure S6: Temperature dependence of K_{eff}, A and Δ. Normalized temperature dependence of the effective anisotropy constant (red), the exchange stiffness constant (blue) and the analytical domain wall width Δ (green).

Using again the temperature scaling of the exchange stiffness constant and the effective anisotropy in combination with the domain wall width w(300 K)= 8nm obtained from the micromagnetic simulations and Eq. (16), leads to the domain wall widths displayed in Fig. S7.

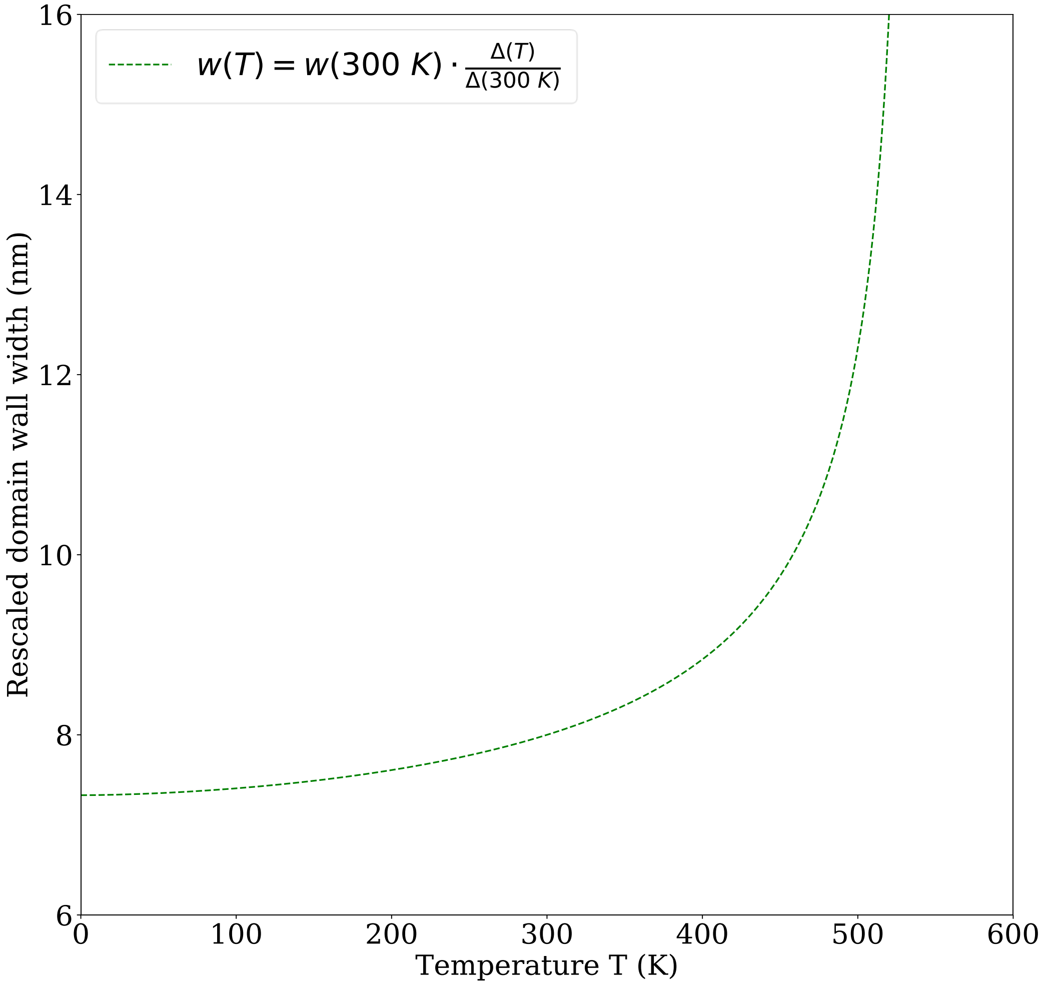

Figure S7: Temperature dependence of w. Temperature dependence of the domain wall width w based on the temperature scaling of the exchange stiffness constant and effective anisotropy.

Combining the time delay dependence of the spin system temperature displayed in Fig. S3 with the here obtained temperature dependence of the domain wall width one can estimate the equilibrium domain wall width at increased temperature for delay times between 1 ps to 100 ps to approximately 11 nm.

S5: Fourier spectrum of the MFM image

The magnetic force microscopy image displayed in Fig. 1b was Fourier transformed leading to the spectrum displayed in Fig. S8. One can see a clear first order peak at a value of q=(13.8+/-0.9) μm^{-1} as well as a second order peak at q=(27.6+/-0.9) μm^{-1} and a third order peak at q=(41.4+/-1.0) μm^{-1} due to partial short range stripe alignment of the OOP demagnetized magnetic labyrinth pattern. The first order peak indicates a stripe periodicity of (455+/-30) nm, which agrees well with the real space stripe data measured.

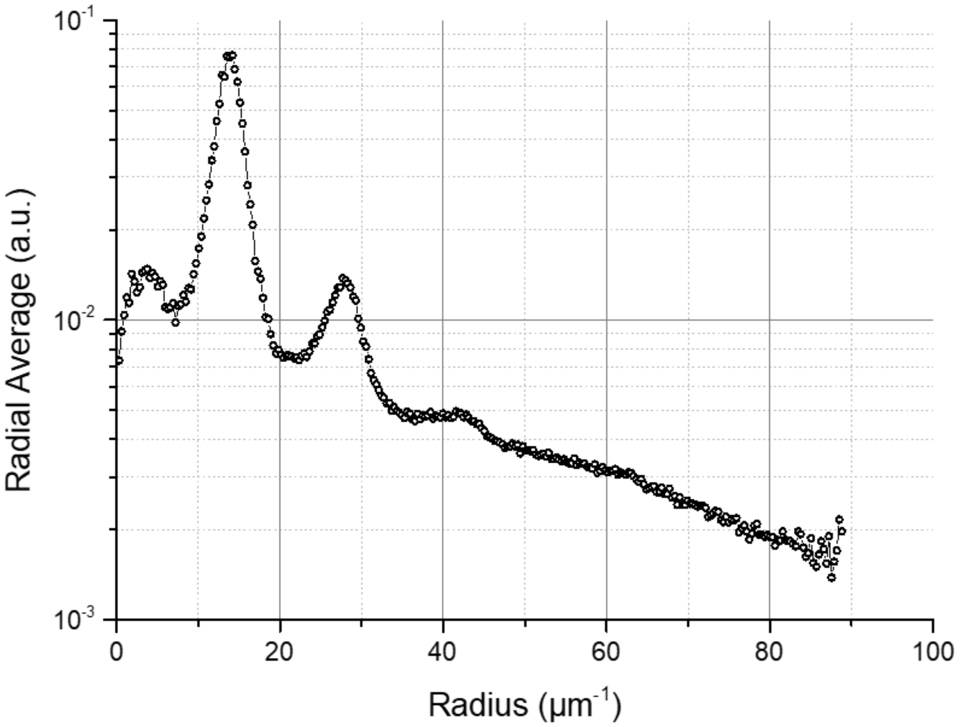

Figure S8: Fourier Spectrum of MFM image. Logarithmic Fourier spectrum of the MFM image of the demagnetized labyrinth stripe state showing a first, second and third order peak.

S6: Fit parameters of the pump-probe delay curves from 0 - 3 ps

	Figure 4a		Figure 4b	
	CL+CR	***CL-CR***	***CL+CR***	***CL-CR***
A_D	-0.80±0.01	-0.85±0.03	-0.86±0.01	-0.89±0.01
τ_D, ps	0.39±0.10	0.31±0.10	0.16±0.01	0.16±0.01
A_{R1}*	*~0.04*	*~0.10*	0.05±0.01	0.05±0.02
A_{R2}*	*~0.76*	*~0.80*	0.78±0.01	0.81±0.02
τ_{R1} *, ps	*~1±1*	*~1±1*	*14±7*	*9±6*
τ_{R2}, ps	>900	312±18	>2000	530±100

*) Some of the refinement parameters are difficult to determine from the fit due to an ill-conditioned Jacobian.

Table S1: Pump-probe fit parameters. Fit parameters obtained by fitting the pump-probe data shown in Fig. 4a & 4b with the model described in Eq. (3).

S7: Ultrafast demagnetisation within the first picoseconds

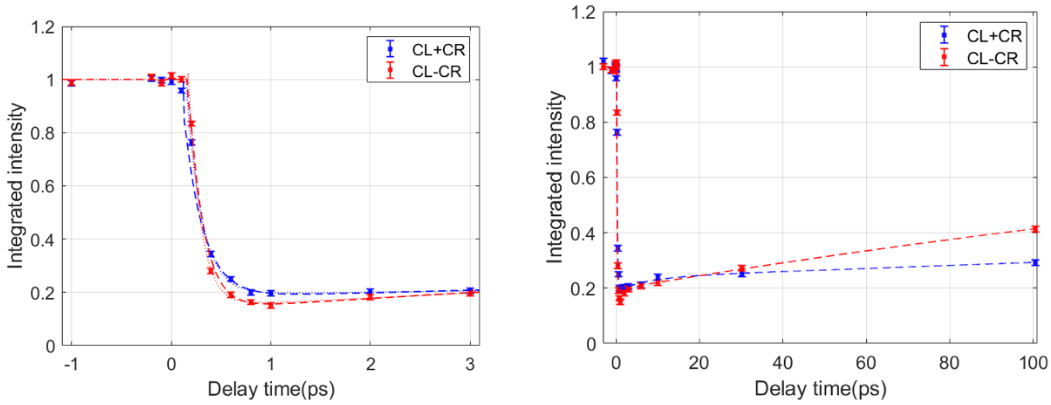

Figure S9: Fits of the pump-probe delay curves. Left: Ultrafast demagnetization (same data as in Fig. 4(a)) - solid lines represents a fit with Eq. (3) in the time interval t = (0-3) ps. Right: Solid line represents a fit over the entire time interval with Eq. (3).

S8: Estimation of error bars

There are the following sources of fluctuations contributing to the error bar:

- Poisson noise from photon counting statistics. The CCD scattering images (see Fig. 1 and 2) represent averages of 7000 FEL pulses each - with the CCD camera read out after the 7000 pulses. The data points shown in Fig. 4a and 4b have been determined by azimuthal and radial averaging over 7.12x10^5 pixels in the relevant Q-range. The counting error based on Poisson statistics is $\Delta I = \sqrt{I}$ yielding a relative error of $\Delta I/I = 1/\sqrt{I}$. The averaged count numbers are rather high in the range of 0.1 to 1x10^6 photons implying relatively small error bars. We find typical values of $\Delta I/I$= 0.3 % at t = 1ps and $\Delta I/I$= 0.2 % at t=100 ps for CL-CR and 0.16 % (1ps) and 0.13% (100 ps) for CL+CR.
- FEL fluctuations. The FERMI FEL is a seeded FEL and intensity fluctuations were relatively small with standard deviation of 10-20 % during the experiment. With 7000 shots the error is std/sqrt(7000) ranging around $\Delta I/I$=0.1- 0.2 %.
- Speckle fluctuations. The use of a highly coherent beam results in a speckle pattern, visible by the grainy appearance of the CCD images and also by the non-flat appearance of the scattering curves in Fig. 3. The fact that they appear always at the same location in reciprocal space demonstrates the speckle origin of these features. The graininess of a speckle pattern can also yield fluctuations in the average intensity with values depending on the number of speckles N and external fluctuations produced by the pointing instability of the FEL (around 1/3 to 1 sigma of beam, FWHM). The reader may think about the extreme case if a single speckle is fluctuating. We estimate the magnitude of this contribution in the following way: The speckle size is given by $S = L\lambda/D$ with L denoting the sample-detector distance, λ the wavelength and D the beam size. The number of speckles in the Q-area of interest between Q_2 and Q_1 is $N = \frac{\pi(L\lambda)^2(Q_2^2-Q_1^2)}{(4\pi)^2S^2}$. From the central part of I(Q) with Q=14.00 to 15.00 μm^{-1} we obtain N=1000 and thus estimate an upper bound for a statistical fluctuation of $\Delta N/N\sim 0.65$ %.

Combining all sources of fluctuations result in the error bars shown in the manuscript with values of around 1%. We can compare this number to the experimental data by calculating the standard deviation of the first five measured data points in the time interval t=-3,-1,-0.2,-0.1,0 ps before pumping (=constant intensity). The standard deviation for CL+CR and CL-CR are 1.3 % each, which is in reasonable agreement with the values calculated above.

S9 Additional Scans

In a second round of experiments we measured with lower scattering intensity three pump-probe scans. The data shown in Fig. 4b represent the average of these three scans.

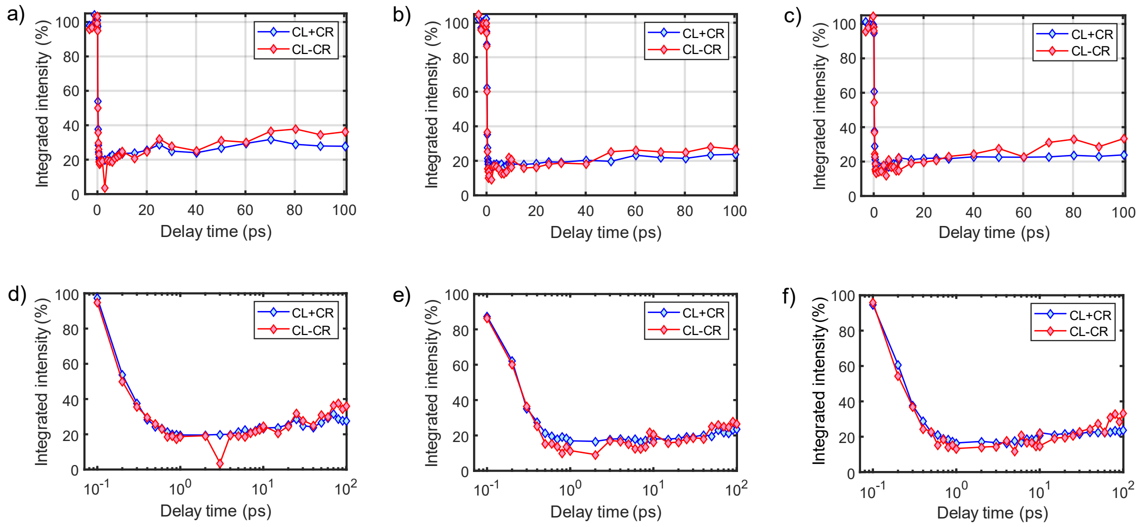

Figure S10: Additional scans including more data points at different times. Three additional scans with lower scattering intensity and more time points.

References

[1] Lemesh, I., Büttner, F., & Beach, G. S. D. Accurate model of the stripe domain phase of perpendicularly magnetized multilayers. *Phys. Rev. B* **95**, 174423 (2017).

[2] https://github.com/MicroMagnum/MicroMagnum

[3] Conte, R. L. et al. Role of B diffusion in the interfacial Dzyaloshinskii-Moriya interaction in Ta/Co20Fe60B20/MgO nanowires. *Phys. Rev. B* **91**, 014433 (2015).

[4] Casiraghi, A. et al. Individual skyrmion manipulation by local magnetic field gradients. *Commun. Phys*. **2**, 145 (2019).

[5] Legrand, W. et al. Hybrid chiral domain walls and skyrmions in magnetic multilayers. *Sci. Adv.* **4**, eaat0415 (2018).

[6] Thiaville, A. et al. Dynamics of Dzyaloshinskii domain walls in ultrathin magnetic films. *EPL* **100**, 57002 (2012).

[7] Atxitia, U. et al. Multiscale modeling of magnetic materials: Temperature dependence of the exchange stiffness. *Phys. Rev. B* **82**, 134440 (2010).
